# The Effectiveness of a Resilience Enhancement Program on the Quality of Life Among Adolescents with Thalassemia in Nakhon Si Thammarat Province, Thailand

**DOI:** 10.3390/healthcare14091184

**Published:** 2026-04-28

**Authors:** Yothaka Meelong, Kiatkamjorn Kusol, Thidarat Eksirinimit

**Affiliations:** 1 Thasala Hospital, Nakhon Si Thammarat 80160, Thailand; yothaka99.m@gmail.com; 2 School of Nursing, the Excellence Center of Community Health Promotion, Walailak University, Nakhon Si Thammarat 80160, Thailand; athidara@gmail.com

**Keywords:** resilience, quality of life, adolescents with thalassemia

## Abstract

**Background:** Thalassemia is an inherited hemoglobin disorder and is among the most prevalent genetic diseases worldwide. In adolescents, the physical consequences of thalassemia extend beyond physiological impairment and affect daily functioning, education, and body image. **Objective:** This research aimed to promote resilience and quality of life among adolescents with thalassemia, with the goal of increasing mean resilience and quality-of-life scores before and after participation. Methods: This cluster quasi-experimental study employed a two-group pretest–posttest design with a sample of 58 adolescents aged 12–17 years diagnosed with thalassemia. Participants were allocated to the experimental (n = 29) and control (n = 29) groups using a cluster-based approach at the district level. The intervention lasted 8 weeks. The assessment tools included the Pediatric Quality of Life Inventory and the Resilience Scale. Data were analyzed using descriptive statistics, including frequency distributions, percentages, means, standard deviations, t-tests, chi-square tests, and Fisher’s exact tests. **Results:** Baseline comparisons indicated no statistically significant differences in personal characteristics between the two groups (*p* > 0.05). Before the intervention, the mean resilience scores in both groups were low. The mean quality-of-life scores for the experimental and control groups were moderate. After participating in the resilience enhancement program, the experimental group showed statistically significant increases in both resilience and quality-of-life scores relative to pre-intervention levels (*p* < 0.001). Additionally, these post-intervention scores were significantly higher than those of the control group (*p* < 0.001). **Conclusions:** These findings indicate that the resilience enhancement program effectively improved resilience and enhanced the quality of life among adolescents living with thalassemia.

## 1. Introduction

Thalassemia is an inherited hemoglobin disorder and represents one of the most prevalent genetic diseases worldwide. Due to its high global burden, long-term morbidity, and substantial healthcare demands, thalassemia has been recognized by the World Health Organization as a significant public health concern [1]. The disorder is particularly prevalent in Southeast Asia, a region characterized by a high carrier frequency of thalassemia genes, influenced by historical genetic distribution and increasing population mobility [2]. Among Asian countries, Thailand ranks fourth in terms of thalassemia gene prevalence, resulting in a sustained and significant burden on the national healthcare system [3]. As a chronic condition requiring lifelong medical management, thalassemia imposes considerable economic, social, and psychological costs at the individual, familial, and societal levels [4].

Adolescents with thalassemia experience fundamental abnormalities in hemoglobin synthesis, leading to chronic anemia, increased red blood cell destruction, and ineffective erythropoiesis. These pathophysiological processes contribute to a wide range of clinical manifestations, including hepatosplenomegaly, delayed physical growth, skeletal deformities, and characteristic facial changes [5]. Continuous blood transfusions and long-term treatment are often required to manage disease progression and prevent complications. Consequently, the physical consequences of thalassemia extend beyond physiological impairment and significantly affect daily functioning, self-perception, and body image, particularly during adolescence, a developmental period in which physical appearance and social acceptance play critical roles [6].

The quality of life of adolescents with thalassemia has been found to be significantly affected by academic problems, primarily due to frequent school absences for hospital treatment, which result in a noticeable decline in academic performance [7]. In the educational domain, adolescents with thalassemia have been reported to have a low quality of life [8,9]. In addition, these adolescents often experience anemia, underweight status, delayed growth, and skeletal deformities from bone changes. Patients may also present hepatomegaly and splenomegaly, along with characteristic skeletal alterations [10]. Moreover, they exhibit distinctive facial features, including prominent cheekbones and a widened interorbital distance (commonly referred to as thalassemia facies)*,* as well as splenomegaly [11]. These conditions collectively have a significant impact on the body image of adolescents with thalassemia.

Psychological resilience has emerged as a key protective factor, enabling individuals to cope effectively with adversity, stress, and chronic health conditions [12]. In adolescents with chronic illnesses such as thalassemia, resilience plays a pivotal role in facilitating emotional regulation, adaptive coping, and psychological recovery. Importantly, resilience is not a fixed trait but a dynamic capacity that can be strengthened through targeted interventions and structured psychosocial programs. Previous studies have demonstrated that resilience-enhancing interventions can significantly reduce stress levels while improving resilience and overall quality of life among adolescents with thalassemia [13,14]. Additionally, when adolescents receive psychological resilience-enhancing interventions, they show improvements in cognitive processes, perspectives, attitudes, coping strategies, problem-solving skills, and self-development, thereby increasing psychological resilience [15]. Studies examining the effectiveness of psychological resilience development programs among adolescents have shown that early adolescents who participated in such programs exhibited significantly higher resilience scores than those who did not [16]. The development of psychological resilience based on the conceptual framework proposed by Grotberg consists of three core components: (1) “I Am,” which helps adolescents gain greater self-awareness and self-understanding; (2) “I Can,” which enables adolescents to understand problems, identify appropriate solutions, and manage challenges creatively; and (3) “I Have,” which promotes adolescents’ understanding of their surrounding environment, sense of responsibility, and the development of positive social relationships [17]. Evidence from prior studies indicates that adolescents who participate in psychological resilience development programs demonstrate higher resilience levels than those who do not, along with improvements in analytical thinking, coping skills, perseverance, self-esteem, life goals, and personality development [18,19].

Research on stress management and resilience training among patients with thalassemia, particularly through breathing-based techniques, has shown reductions in stress levels and improvements in psychological resilience and quality of life [20]. A review of the literature reveals that most studies have focused on the parents of children with thalassemia and on the development of psychological resilience in children and adolescents, while relatively few have examined the combined impact of resilience enhancement programs on quality of life, particularly among adolescents with chronic illnesses such as thalassemia in the Thai context. Adolescents with thalassemia experience substantial impairments in physical, psychological, social, and academic domains of quality of life. As a community nurse practitioner, the researcher recognizes the importance of strengthening adolescents’ psychological resilience in the context of chronic thalassemia to improve quality of life.

This study included adolescents diagnosed with thalassemia residing in four semi-urban and rural districts. Therefore, implementing a resilience-enhancing program that is not complex and does not require extensive time for activities in the hospital setting—while participants attend scheduled outpatient visits and receive blood transfusions—as well as incorporating home visits, may increase opportunities for participants to develop skills and learn cognitive approaches that contribute to strengthening resilience and improving their quality of life. This research aims to design a program to promote resilience and quality of life among adolescents with thalassemia, with the research hypothesis that the mean quality-of-life scores will increase from before to after participation. Additionally, the post-intervention quality-of-life means scores in the experimental group would be higher than those in the control group. The program incorporates structured activities to enhance psychological resilience, promote self-care, improve social competence, foster creative problem-solving, encourage life-goal orientation, and support adolescents in managing their chronic illness while maintaining a good quality of life.

## 2. Materials and Methods

### 2.1. Study Design

This study used a cluster quasi-experimental research design with two groups and pre- and post-tests. The sample consisted of adolescents aged 12–17 years who had been medically diagnosed with thalassemia in Nakhon Si Thammarat Province, Thailand. Data were collected from August to November 2024.

### 2.2. Measures

The sample size was calculated using G*Power 3.1.9.7 [Heinrich Heine University Düsseldorf, Düsseldorf, Germany] based on effect size estimates derived from a previous study on the effects of a psychological resilience-enhancing program among early adolescents in a school in Songkhla Province conducted by Kanda Nakwaree, Patcharin Nintachan, and Sophin Saeng-on [21]. This study employed a quasi-experimental two-group pretest–posttest design. The sample size for the present study was determined based on the outcomes reported in that study. Specifically, the experimental group’s mean score was 113.28, while the control group’s was 104.31. The control group’s standard deviation was 9.17. These values were used to calculate the effect size, which was 0.97. In determining the sample size, the researchers estimated the effect size to be 0.97 at a 95% confidence level (α = 0.05) with 95% power, resulting in 29 participants per group. To account for potential attrition, the target sample size was increased by 10% to 32 participants per group. During the study period, three participants in each group were lost to follow-up, resulting in a final sample of 29 participants in each group.

A cluster-based allocation procedure was used at the district level. Four districts were selected from the 23 districts in Nakhon Si Thammarat Province: Thasala, Sichon, Thung Song, and Chian Yai. Districts were then assigned to the experimental or control groups. Thasala and Sichon were allocated to the experimental group, whereas Thung Song and Chian Yai were allocated to the control group. This design was selected to minimize contamination among participants, given the group-based nature of the intervention. Participants were then recruited within each district according to the inclusion and exclusion criteria.

The inclusion criteria were as follows: (1) aged 12–17 years and diagnosed with thalassemia by a physician; (2) able to communicate in Thai, including speaking, reading, and writing; (3) not currently participating in another study related to quality of life or psychological resilience; (4) no severe comorbid conditions requiring concurrent treatment, such as heart disease, kidney disease, or autoimmune disease; and (5) both adolescent assent and parental or guardian consent were obtained.

The exclusion criteria were the inability to participate in the research activities as scheduled and the development of disease-related complications during the study period.

### 2.3. Research Instruments

1. Questionnaire on general information of adolescents aged 12–17 years (i.e., sex, age, religion, education level, duration of illness, caregiver, and education level of caregiver)
2. The Pediatric Quality of Life Inventory consists of 23 items across four domains: Physical Functioning (8 items), Emotional Functioning (5 items), Social Functioning (5 items), and School Functioning (5 items). Items are rated on a 5-point scale and transformed to a score ranging from 0 to 100, with higher scores indicating better quality of life. In this study, the instrument was adapted from the study examining factors influencing quality of life among pediatric patients with thalassemia [22]. The instrument was pilot-tested among 30 adolescents with characteristics similar to those of the study sample, yielding a Cronbach’s alpha of 0.82. Quality of life scores were categorized into four levels based on the following criteria:

0.00–25.00Very low level26.00–50.00Low level51.00–75.00Moderate level76.00–100.00High level

3. The Resilience Scale for Children and Adolescents, developed by the Department of Mental Health (2020), consists of 48 items covering four domains, with 12 items in each domain: Self-care and self-regulation ability, social competence, problem-solving ability, and life purpose and goal setting [12]. Each item is rated on a 3-point Likert-type scale. In this study, the instrument was pilot tested among 30 adolescents with characteristics similar to those of the study sample, yielding a Cronbach’s alpha coefficient of 0.82. Total and domain scores are categorized into three levels—low, moderate, and high—based on established scoring criteria.

0.00–59.00Low level60.00–80.00Moderate level81.00–96.00High level

4. The resilience enhancement program aimed at improving the resilience and quality of life of adolescents with thalassemia was developed based on the resilience framework proposed by the Department of Mental Health [2020] [12]. The intervention was implemented over an eight-week period and focused on four core domains: (1) Self-care and self-regulation ability (Autonomy). It refers to a sense of autonomy characterized by self-regulation, grounded in the belief that one can control various aspects of one’s life. It encompasses self-esteem, a sense of personal pride, and self-discipline, (2) Social competence. It comprises communication skills, empathy—defined as the ability to understand and care for others—and the capacity to establish and maintain interpersonal relationships, (3) Problem-solving ability, and (4) Sense of meaning and purpose. It focuses on enhancing life motivation and goal-setting, enabling individuals to achieve their personal goals. The program was delivered using the researchers’ handbook, which included knowledge about thalassemia in adolescents, detailed procedures, and structured intervention activities. These nursing roles encompass providing support and care, fostering psychological empowerment, and conducting ongoing supervision and follow-up to ensure the continuous development of psychological resilience in adolescents with thalassemia. The handbook was content-validated by five experts, with an Index of Item-Objective Congruence of 0.87.

The intervention comprised six sessions:

Session 1 (Preparation): Baseline assessment was conducted during routine hospital visits. Participants completed the pre-intervention questionnaires, which took approximately 45–60 min. Appointments were then scheduled for the intervention sessions.

Session 2 (Week 1, hospital-based, small group, 60 min): Activities focused on thalassemia knowledge, self-care, and emotional awareness. Participants were encouraged to ask questions, share experiences, and reflect on emotional regulation.

Session 3 (Week 2, Zoom-based, small group, 45 min): Activities reviewed self-care and self-regulation practices, identified barriers, and encouraged collaborative problem-solving.

Session 4 (Week 4, hospital-based, small group, 60 min): The “Positive Communication with Others” activity aimed to strengthen social skills, constructive communication, and positive expression through reflection and scenario-based exercises.

Session 5 (Week 6, home visit, individual, 45–60 min): The “Life Pathway” activity was designed to promote problem-solving, life purpose, motivation, and goal setting through guided reflection on significant life experiences and future aspirations.

Session 6 (Week 8, hospital-based, 60 min): Participants completed the post-intervention resilience and quality-of-life assessments during routine appointments.

### 2.4. Data Collection

After the research proposal was approved by the Human Research Ethics Committee of Walailak University, permission to conduct the study was formally obtained from the relevant institutions. The study was approved to proceed by the Human Research Ethics Committee, Walailak University (Certificate Number WUEC-24-267-01). The researchers then met with eligible participants and their parents or legal guardians to seek cooperation. The purpose of the study, research procedures, and duration of participation were clearly explained. Written informed consent was obtained from both the children and their parents or guardians prior to participation. The intervention lasted 8 weeks.

In the 1st week, we collected pre-test data from both the control and experimental groups. Week 8 participants completed structured questionnaires covering demographic characteristics, health-related information, the Pediatric Quality of Life Inventory™, and the resilience assessment.

For the experimental group, the researcher organized structured activities that provided knowledge about thalassemia, self-care practices to support appropriate health behaviors, and recognition of abnormal signs and symptoms that require monitoring. Participants were encouraged to share their experiences with emotional regulation and self-care, and to acknowledge and highlight peers who demonstrated effective emotional regulation skills. In addition, the intervention included activities to enhance social competence, communication skills, goal-setting, commitment to success, and problem-solving abilities.

All data were collected by trained research assistants under the supervision of the researchers to ensure accuracy and consistency. Standardized measurement procedures were applied throughout the study to minimize measurement bias and enhance data reliability.

### 2.5. Ethical Statement

All activities involving human participants conducted in the studies complied with the ethical standards outlined in the Ethical Institutional Considerations. This research was approved by the Ethics Committee in Human Research at Walailak University (Certificate Number WUEC-24-267-01), certified on 25 July 2024. The researchers conducted the study in accordance with the Declaration of Helsinki. The researchers contacted the participants to discuss the study’s purpose, significance, and procedures, and explained voluntary participation and the principle of unaffected withdrawal. Before starting, the parent or guardian and participants signed written informed consent. All procedures involving human participants conducted in the studies complied with the ethical standards of the Institutional Review Committee. Participants’ data were only used for academic purposes and will not be disclosed to anyone other than the relevant researchers.

### 2.6. Statistical Analysis

Statistical analyses were performed using IBM SPSS Statistics 26.0.0.1 (Armonk, NY, USA). The data were analyzed using descriptive statistics to summarize baseline general information. Categorical variables are presented as frequencies and percentages, whereas continuous variables are expressed as means and standard deviations. Baseline differences between the experimental and control groups were examined using the Chi-square test or Fisher’s exact test for categorical variables, and the independent samples t-test for continuous variables. The mean scores for resilience and quality of life among adolescents with thalassemia between the control and experimental groups were analyzed using the independent t-test. The mean scores for resilience and quality of life before and after the intervention were compared within the control and experimental groups using paired-samples t-tests. Prior to analysis, the researchers assessed the dataset to verify that it satisfied the fundamental assumptions underlying the statistical methods to be applied.

## 3. Results

The participants’ characteristics showed that most in the experimental group were female (62.07%), with a mean age of 14.07 years (SD = 1.81). Most participants were Buddhist (89.66%). More than half (51.72%) were studying at the lower secondary level. The primary caregivers were predominantly parents (79.31%), followed by uncles or aunts (13.79%). In the control group, more than half of the participants were female (55.17%), with a mean age of 14.41 years (SD = 1.68). Most participants were Buddhist (93.10%). The participants were studying at the lower secondary level (48.28%). Most primary caregivers were parents (89.66%). All participants had been living with thalassemia for more than 10 years (100%). When comparing adolescents with thalassemia, the general information between the experimental and control groups showed no statistically significant differences (*p* > 0.05; Table 1).

Baseline comparisons between the experimental and control groups showed no statistically significant differences in resilience or quality-of-life scores prior to the intervention. The mean resilience score was 43.10 (SD = 4.53) in the experimental group and 45.14 (SD = 4.52) in the control group (t = −1.71, *p* = 0.093, 95% CI [−4.42, 0.35], d = −0.45). The mean quality-of-life score was 71.55 (SD = 3.76) in the experimental group and 69.90 (SD = 3.26) in the control group (t = 1.78, *p* = 0.079, 95% CI [−1.99, 3.51], d = 0.47). These findings suggest that the two groups were comparable at baseline (Table 2).

A comparison of the mean resilience and quality-of-life scores after the intervention showed that the experimental group had significantly higher resilience scores than the control group (M = 75.66 ± 6.07 vs. 46.24 ± 4.23; t = 21.40, *p* < 0.001, 95% CI [26.66, 32.17], d = 5.62). Similarly, post-intervention quality-of-life scores were significantly higher in the experimental group than in the control group (83.69 ± 4.16 vs. 71.28 ± 3.83; t = 11.80, *p* < 0.001, 95% CI [10.31, 14.52], d = 3.10). These effect sizes indicate extremely large between-group differences and strong practical significance (see Table 3).

Within the experimental group, resilience increased significantly from pretest to posttest (t = −29.67, *p* < 0.001, d = 5.50), and quality of life also improved significantly (t = −13.91, *p* < 0.001, d = 2.58). In contrast, no statistically significant changes were observed in the control group for resilience (t = −2.05, *p* = 0.058, d = 0.38) or quality of life (t = −1.92, *p* = 0.065, d = 0.36). Overall, these findings indicate that the resilience-enhancing program had a substantial impact on both resilience and quality of life among adolescents with thalassemia. The results are presented as shown in Table 4.

## 4. Discussion

This study evaluated the effectiveness of a resilience-enhancing program in improving resilience and quality of life among adolescents with thalassemia aged 12–17 years in Nakhon Si Thammarat Province, Thailand. The findings support the research hypothesis. First, adolescents in the experimental group demonstrated significant improvements in both resilience and quality of life after participating in the program. Second, adolescents in the experimental group showed significantly higher resilience and quality of life after participating in the program than the control group.

The intervention focused on enhancing autonomy, social competence, problem-solving skills, and a sense of meaning and purpose. These components may have contributed to improved emotional self-regulation, strengthened social support networks, enhanced analytical thinking, and the establishment of future-oriented goals [12]. Adolescents with thalassemia in the experimental group showed a significantly higher mean resilience score after participating in the program. The program may have helped adolescents cope with developmental challenges and illness by enabling effective stress management and emotional regulation, thereby improving psychological well-being. Nevertheless, resilient adolescents are capable of positive adaptation and can effectively cope with illness-related challenges [19,23]. Health care providers should collaborate to improve their resilience [24]. Because anxiety is common among adolescent thalassemia survivors, body image, self-esteem, and coping skills are key factors. The need for psychological support focused on body image, self-esteem, and coping strategies to improve mental health among adolescent thalassemia [25]. These findings are consistent with previous research suggesting that resilience can be enhanced through structured psychosocial interventions [16,18,19]. Prior studies have also shown that improved resilience is associated with better coping, emotional adjustment, and psychological well-being among adolescents living with chronic conditions such as thalassemia [20,23,24].

Moreover, these results align with our study, showing that students’ mean life resilience scores increased after participating in the program. Furthermore, after the intervention, the experimental group had significantly higher mean life-resilience scores than the control group. The findings suggest that the program can be applied to promote life resilience among lower secondary school students, thereby enhancing their preparedness to cope with future life crises [21].

Importantly, the effect sizes observed in this study ranged extremely large, indicating not only statistical significance but also substantial practical significance. The intervention was structured into six sessions delivered through hospital-based activities, online follow-up, and home visits. The findings suggest that the intervention may have had a meaningful impact on participants’ psychosocial functioning and daily life. Possible explanations include the relatively low baseline scores, the intensive nature of the program, and the close alignment of the intervention content with the psychosocial needs of adolescents with thalassemia.

The significant improvement in quality of life in the experimental group may also be explained by the resilience framework underlying the intervention. The quality of life can be improved through building psychological resilience, as it strengthens internal psychological resources that may help adolescents adapt more effectively to stress, regulate emotions, engage positively with others, and develop a more positive self-conception, which ultimately improves daily life among adolescents with thalassemia [20,26]. These findings are consistent with research showing that adolescents demonstrated enhanced self-esteem and a more resilient, well-integrated personality [17,27]. Such skills are particularly important for adolescents with thalassemia, who face long-term treatment demands and psychosocial stress. Through these mechanisms, participants were empowered to actively manage their condition and adapt positively to their quality of life [13,15,28]. Previous studies have similarly reported that social skills training plays a critical role in enhancing psychological resilience by fostering greater optimism, knowledge, and adaptive coping, and thereby improving quality of life [14,19,20]. A previous study found that individuals who received blood transfusions had lower QoL than those who did not [29]. Therefore, healthcare teams involved in pediatric care should implement strategies to enhance resilience during blood transfusion sessions, thereby improving the quality of life of these adolescents. Furthermore, previous research assessed the quality of life (QoL) in the physical and psychological domains among school-age children with major thalassemia compared to healthy peers. The results demonstrated that poor QoL was more prevalent in the thalassemia group than in their healthy counterparts, particularly in the physical and psychological domains. Therefore, continuous monitoring and comprehensive support from health care providers, families, and society, including educational institutions, are essential to prevent deterioration in quality of life and to enhance the well-being of these children [30].

Despite these promising findings, these limitations should be acknowledged. First, the study used a cluster quasi-experimental design, which limits the strength of causal inference compared with a fully randomized controlled trial. Second, the district-level cluster-based allocation may have introduced clustering effects that were not fully accounted for in the analysis. Third, the sample size was relatively small and drawn from a specific geographic area, which may limit the generalizability of the findings. Fourth, the use of self-reported measures may have introduced response bias. Finally, the follow-up period ended at the end of the intervention and did not allow evaluation of longer-term effects. Future studies should include larger samples, longer follow-up periods, and more advanced analytical approaches, such as mixed-effects models, to better account for cluster-level variation and confirm the robustness of the findings.

## 5. Conclusions

The resilience-enhancing program demonstrated significant effectiveness in improving adolescents’ psychosocial outcomes with thalassemia. Participation in the resilience-enhancing program led to a significant increase in both resilience and quality of life among adolescents. As a result, the resilience-enhancing program served as a structured psychosocial intervention, equipping adolescents with essential coping skills, emotional regulation, and social resources. This transformation enabled participants to shift from passive recipients of illness-related stress to active, resilient individuals, as reflected in significantly improved resilience and quality-of-life outcomes.

## Figures and Tables

**Table 1:** General information on adolescents with thalassemia.

Data	Total (n = 58)	Control Group (n = 29)	Experimental Group (n = 29)	*p*-Value
	Number (%)	Number (%)	Number (%)	
Gender				0.59 ^a^
Female	34 (58.62%)	16 (55.17%)	18 (62.07%)	
Male	24 (41.38%)	13 (44.83%)	11 (37.93%)	
Age (12–17 years old)	Mean = 14.24 (SD = 1.74)	Mean = 14.41 (SD = 1.68)	Mean = 14.07 (SD = 1.81)	0.82 ^c^
Religion				0.64 ^b^
Buddhist	53 (91.38%)	27 (93.10%)	26 (89.66%)	
Islam	5 (8.62%)	2 (6.90%)	3 (10.34%)	
Education				0.41 ^a^
Primary Education	14 (24.14%)	9 (31.03%)	5 (17.24%)	
Lower Secondary Education	29 (50.00%)	14 (48.28%)	15 (51.72%)	
Upper Secondary Education	15 (25.86%)	6 (20.69%)	9 (31.03%)	
Caregiver				0.37 ^b^
Parents	49 (84.48%)	26 (89.66%)	23 (79.31%)	
Grandparents	4 (6.90%)	2 (6.90%)	2 (6.90%)	
Uncle/Aunt	5 (8.62%)	1 (3.45%)	4 (13.79%)	
Duration of illness				1.00 ^a^
>10 years	58 (100.00%)	29 (100.00%)	29 (100.00%)	

*p* > 0.05, ^a^ = Chi-square, ^b^ = Fisher’s exact test, ^c^ = *t*-test.

**Table 2:** Comparison of pre-intervention resilience and quality-of-life scores in adolescents with thalassemia between groups (n = 58).

Data	Experimental Group (n = 29)		Control Group (n = 29)		Mean Difference	df	t	*p*-Value	95% *CI*		*d*
	M	SD	M	SD					Lower	Upper	
Resilience	43.10	4.53	45.14	4.52	−2.03	56	−1.71	0.093	−4.42	0.35	−0.45
Quality of Life	71.55	3.76	69.90	3.26	1.66	56	1.78	0.079	−1.99	3.51	0.47

*p* > 0.05, df = degree of freedom, *d* = Cohen’s d.

**Table 3:** Comparison of post-intervention resilience and quality-of-life scores in adolescents with thalassemia between groups (n = 58).

Data	Experimental Group (n = 29)		Control Group (n = 29)		Mean Difference	df	t	*p*-Value	95% *CI*		*d*
	M	SD	M	SD					Lower	Upper	
Resilience	75.66	6.07	46.24	4.23	29.41	56	21.40	0.001 ***	26.66	32.17	5.62
Quality of Life	83.69	4.16	71.28	3.83	12.41	56	11.80	0.001 ***	10.31	14.52	3.10

*p* < 0.001 ***, df = degree of freedom, *d* = Cohen’s d.

**Table 4:** Comparison of resilience and quality-of-life data in adolescents with thalassemia before and after intervention within the experimental and control groups (n = 58).

Resilience and Quality of Life	Before		After		t	*p*-Value	95% *CI*		*d*
	M	SD	M	SD			Lower	Upper	
Resilience									
Experimental (n = 29)	43.10	4.53	75.66	6.07	−29.67	0.001 ***	−34.80	−30.31	5.50
Control (n = 29)	45.14	4.52	46.24	4.23	−2.05	0.058	−2.21	0.002	0.38
Quality of Life									
Experimental (n = 29)	71.55	3.76	83.69	4.16	−13.91	0.001 ***	−13.92	−10.35	2.58
Control (n = 29)	69.90	3.26	71.28	3.83	−1.92	0.065	−2.85	0.092	0.36

*p* < 0.001 ***, df = degree of freedom, *d* = Cohen’s d.

## Data Availability

The data presented in this study are available on request from the corresponding author due to privacy reasons.
